# Implementation of evidence-based primary cancer prevention interventions in MA community health centers: an explanatory sequential mixed methods study

**DOI:** 10.21203/rs.3.rs-2588180/v1

**Published:** 2023-02-20

**Authors:** REBEKKA M LEE, James G Daly, Kamini Mallick, Shoba Ramanadhan, Cristina Huebner Torres, Cassidy R Hayes, Alyssa Manuel, Ra’Shaun Nalls, Karen M Emmons

**Affiliations:** Harvard University T H Chan School of Public Health; Harvard University T H Chan School of Public Health; Harvard University T H Chan School of Public Health; Harvard University T H Chan School of Public Health; Caring Health Center; Caring Health Center; Caring Health Center; Harvard University T H Chan School of Public Health; Harvard University T H Chan School of Public Health

**Keywords:** Nutrition, physical activity, tobacco, Federally Qualified Health Centers, community, partnership, cancer prevention

## Abstract

**Background:**

More than half of cancers could be prevented by employing evidence-based interventions (EBIs), including prevention interventions targeting nutrition, physical activity, and tobacco. Federally qualified health centers (FQHCs) are the primary source of patient care for over 30 million Americans – making them an optimal setting for ensuring evidence-based prevention that advances health equity. The aims of this study are to: 1) determine the degree to which primary cancer prevention EBIs are being implemented within Massachusetts FQHCs and 2) describe how these EBIs are implemented internally and via community partnerships.

**Methods:**

We used an explanatory sequential mixed methods design to assess the implementation of cancer prevention EBIs. First, we used quantitative surveys of FQHC staff to determine the frequency of EBI implementation. We followed up with qualitative one-on-one interviews among a sample of staff to understand how the EBIs selected on the survey were implemented. Exploration of contextual influences on implementation and use of partnerships was guided by the Consolidated Framework for Implementation Research (CFIR). Quantitative data were summarized descriptively, and qualitative analyses used reflexive, thematic approaches, beginning deductively with codes from CFIR, then inductively coding additional categories.

**Results:**

All FQHCs indicated they offered clinic-based tobacco interventions, such as clinician-delivered screening practices and prescription of tobacco cessation medications. Quitline interventions and some diet/physical activity EBIs were available at all FQHCs, but staff perceptions of penetration were low. Only 38% of FQHCs offered group tobacco cessation counseling and 63% referred patients to mobile phone-based cessation interventions. We found multilevel factors influenced implementation across intervention types – including the complexity of intervention trainings, available time and staffing, motivation of clinicians, funding, and external policies and incentives. While partnerships were described as valuable, only one FQHC reported using clinical-community linkages for primary cancer prevention EBIs.

**Conclusions:**

Adoption of primary prevention EBIs in Massachusetts FQHCs is relatively high, but stable staffing and funding are required to successfully reach all eligible patients. FQHC staff are enthusiastic about the potential of community partnerships to foster improved implementation - providing training and support to build these relationships will be key to fulfilling that promise.

## Background

More than half of cancers could be prevented by employing existing evidence-based interventions (EBIs), including prevention interventions targeting nutrition, physical activity, and tobacco (1). Serving as the primary source of patient care for over 30 million Americans, (2) Federally Qualified Health Centers (FQHCs) funded by the Health Resources and Services Administration (HRSA) are well-positioned to reach populations experiencing health disparities with evidence-based cancer prevention interventions.

However, little is known about how primary cancer prevention EBIs are being implemented by FQHCs. Research has demonstrated successful implementation of cancer screening interventions in FQHCs (3–5) and HRSA now includes tobacco screening and cessation counseling as a quality metric (2). Yet, understanding the usage of evidence-based behavioral interventions addressing nutrition, physical activity, and tobacco use, which have the potential for prevention of cancer and other chronic diseases, is more limited. Clinical-community linkages have been lifted up as promising practices for chronic disease prevention within research (6–9) and the Agency for Healthcare Research and Quality as they build trust and promote access to disease prevention and treatment to “improve care and support patients better than either of these sectors could do alone” (10). The Centers for Disease Control and Prevention (CDC) presents a continuum of clinical-community linkages ranging from networking for information exchange, to coordinating to increase accessibility to services, to cooperating to share resources, to collaborating to enhance each other’s capacity, to merging to operate as one entity (11, 12). These relationships between clinical and community settings commonly include creating referrals, partnering to deliver clinical services, and cooperating to provide wraparound services that address social determinates of health (13). However, research has not documented what these partnerships entail in real world practice or how they employ EBIs. The aims of this study are to: 1) determine the degree to which primary cancer prevention EBIs are being implemented within Massachusetts FQHCs; and 2) describe how these EBIs are implemented, both internally and via community partnerships.

## Methods

### Design and Setting

This study is based at the Implementation Science Center for Cancer Control Equity (ISCCCE), a National Cancer Institute-funded center with collaboration between the Massachusetts League of Community Health Centers (Mass League), Harvard T.H. Chan School of Public Health, Massachusetts General Hospital, and Dana Farber Cancer Institute. The Mass League is the Primary Care Association that provides workforce development, policy analysis, information technology development, clinical quality improvement (QI), training, and education to 52 FQHCs across Massachusetts (14). The core ISCCCE structure includes an Implementation Lab that is responsible for building the research capacity of 31 FQHCs that share a common population management platform and for supporting engagement in pilot studies.

We used an explanatory sequential mixed methods design to assess the implementation of cancer prevention EBIs in FQHCs (15). The study began with a quantitative survey of staff to determine the frequency with which established EBIs are being implemented. These items were embedded in a broader organizational survey that assessed aspects of the inner setting, outer setting, and characteristics of the individual staff. Following the survey, we conducted qualitative one-on-one interviews with a sub-sample of staff to explore how the EBIs selected on the survey were implemented. The study was approved by the Harvard Longwood Campus Institutional Review Board. The Good Reporting of a Mixed Methods Study Checklist was used to ensure study rigor.

The study was guided by two implementation science frameworks. First, Proctor’s Model for Implementation Outcomes (16), describes the core implementation outcomes that lay on the pathway between interventions and intended clinical patient outcomes. Our survey focused on adoption (e.g., the degree to which Massachusetts FQHCs offer EBIs) and penetration (e.g., the proportion of eligible patients offered or referred to these EBIs). The Consolidated Framework for Implementation Research (CFIR) guided our qualitative exploration (17, 18). This determinants framework describes the potential multilevel contextual influences on EBI implementation, including aspects of the implementation process, characteristics of the intervention itself (e.g., complexity), characteristics of individuals (e.g., staff responsible for delivery), the inner setting (e.g., structures and culture within the FQHC), and the outer setting (e.g., influences outside the FQHC).

### Quantitative Surveys

#### Participants

The ISCCCE team invited staff members from 31 Massachusetts FQHCs to participate. We sampled one to three people within five diverse job type categories – leadership, clinical, QI, community direct-service, or community outreach/engagement. Respondents also self-reported their role on the survey, which includes these categories as well as management, administrative, referrals, consultation, and data support. The survey used branching logic to have respondents who selected community direct-service, community outreach/engagement, or management roles complete items pertaining to EBI implementation given the alignment of their positions to the community partnership aims of this study.

#### Measures

The research team fielded a 10–20-minute online survey via REDCap between November 2020 and August 2021. To ensure culturally appropriate engagement and build rapport with each FQHC, a Mass League leader emailed notification of the survey several days before the invitation was emailed from the ISCCCE principal investigator via REDCap. Up to three reminders to complete the survey were made by email following the initial invitation.

**Figure 1:**
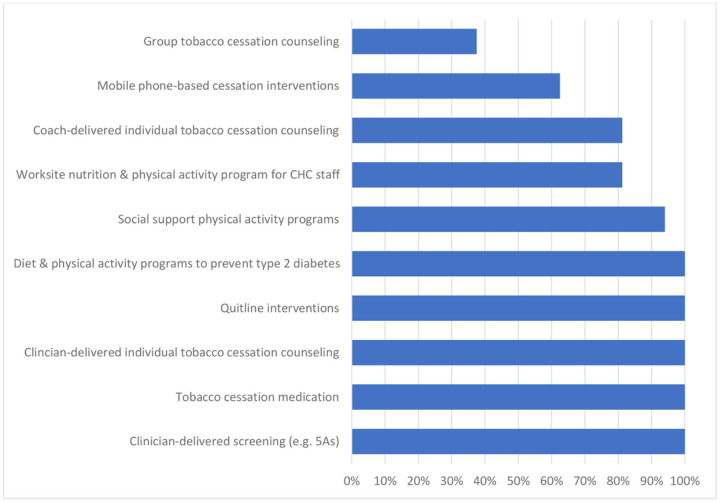
Evidence-based Intervention Adoption at 16 Massachusetts Federally Qualified Health Centers

The survey included close-ended items to identify specific primary prevention EBIs (see Fig. 1) that can be delivered by FQHCs or community-based organizations focused on nutrition, physical activity, and tobacco cessation. We included EBIs that focused on prevention of other chronic diseases (e.g. diabetes) if the primary behavior change targets of intervention were nutrition and physical activity. We included individual or group-delivered EBIs, drawing from The Community Guide (19) as well as input from the research team. Following Proctor’s Model for Implementation Outcomes (16), staff were asked to estimate the proportion of eligible people that are impacted by each intervention (i.e., penetration) on a five-point scale, ranging from “none” to “most or all.” We utilized this self-reported survey measure because it was easily understood by a wide range of FQHC staff with and without prior engagement in research and allowed participants to share their perception of patient impact (vs. yes/no adoption). Surveys also included open-ended questions for participants to describe interventions that were not pre-specified or were “home-grown” (e.g., developed by the FQHC). We also measured participants’ FQHC tenure and demographics (e.g., gender, race/ethnicity, age). Cognitive interviews were conducted with three staff from centers not included in the study sample to review the EBIs included and improve survey language and flow prior to launch of the survey. Respondents were compensated $25 for participation. FQHCs that were new to ISCCCE research projects also received $100 site-level incentives to provide meals or snacks for staff.

#### Analysis

We calculated descriptive statistics for participants’ time in current role and demographics (e.g., race, ethnicity, age, gender), as well as number of eligible people that are offered or referred to the intervention. EBI adoption was summarized at the site level.

### Qualitative Interviews

#### Participants

Researchers invited 23 staff members (1–4/site) from 12 sites to participate in qualitative interviews, beginning with those who completed the survey and then adding interviewee referrals of individuals, such as those in quality improvement and community engagement roles who had in-depth knowledge of FQHC implementation and outreach processes for the types of prevention interventions under investigation. Interviewees included QI and population health leaders, staff managing prevention programming and community engagement activities, and practitioners delivering interventions.

#### Measures

We explored the experience of adoption and implementation of cancer prevention EBIs in greater depth via one-hour, one-on-one semi-structured interviews. Interviews were audio-recorded and then transcribed for accuracy. Researchers asked interviewees to describe how and why each intervention was initially adopted, implemented, and sustained using adoption data from quantitative surveys to structure interview prompts. Participants were also asked whether they implemented other tobacco, nutrition, and physical activity interventions in the past or hoped to in the future. To address Aim 2, participants were asked how partners were involved in implementation of each intervention and where intervention activities were situated (e.g., FQHC or another community setting). Contextual influences were explored using interview probes structured following CFIR to explore multilevel determinants on implementation (17, 18), APPENDIX A). Participants were compensated $50 for participation in interviews.

#### Analysis

We utilized reflexive, thematic approaches, as described by Braun and Clarke (20), beginning deductively with codes from CFIR, then inductively coding additional categories using NVivo Software. To increase credibility and rigor, we utilized researcher triangulation; interviews were coded by the principal investigator and the research project manager, who both have backgrounds conducting public health and implementation science research, to ensure deep engagement with the data and integration of multiple perspectives (21). A third member of the research team, who has a background in medical anthropology, created summaries of codes prioritized for this analysis. Drawing on concepts of information power, we determined that our sample size would be sufficient given fairly broad research goals, a somewhat homogeneous sample (in terms of work focus), rich data collection, and strong reliance on an existing framework (22). Interpretation of results was support by three co-authors who are FQHC staff and researchers with extensive experience in community-based cancer implementation science.

## Results

### Respondents

Of the 146 staff members invited, 71 (49%) completed the survey. Thirty-four staff members from 16 FQHCs selected community direct-service, community outreach/engagement, or management roles and subsequently responded to the questions of focus for this investigation. Of the 16 FQHCs, 13 are based in cities and three in suburban/rural areas. Over 87% of these community-focused staff respondents identified as women. Approximately 56% identified as White, followed by Hispanic/Latino (21%), Asian (15%), and Black/African American (6%). Respondents ranged in age from 27 to 63 years (mean 41). Tenure ranged widely from under one year to 27 years of experience, with an average of 7 years in the current position. Twenty-three staff members were purposively sampled to participate in qualitative interviews. Of those invited, 12 (52%) staff from eight FQHCs participated in follow-up qualitative interviews.

### Quantitative Surveys

Self-reported adoption and penetration of nutrition, physical activity, and tobacco cessation EBIs at Massachusetts FQHCs appear in Fig. 1 and Table 1. All FQHCs indicated they offered clinic-based tobacco interventions, such as clinician-delivered screening practices, individual tobacco cessation counseling, and prescription of tobacco cessation medications. Quitline interventions (e.g., QuitWorks) were available at all FQHCs, but staff perceived they were not offered to many or most eligible smokers. Additionally, tobacco cessation interventions that relied on collaboration outside of the clinical visit were not implemented consistently. Only 38% of FQHCs offered group tobacco cessation counseling and 63% referred patients to mobile phone-based cessation interventions (e.g., SmokefreeTXT).

Evidence-based diet and physical activity programs to prevention type 2 diabetes were offered at all FQHCs. However, only 57% of respondents perceived that many or most eligible patients were offered these programs. When prompted to name other programs offered, some were evidence-based (e.g., Chronic Disease Self-Management Program), but many appeared to be “homegrown” with limited evidence of effectiveness.

### Qualitative Interviews

Our qualitative findings are organized by intervention type (tobacco cessation, nutrition and physical activity) and CFIR domain (implementation process, intervention characteristics, inner setting, characteristics of individuals, outer setting). We also describe how partnerships with community organizations were employed in EBI implementation.

### Tobacco Cessation

#### Implementation Process

Staff reported conducting tobacco screening annually or more frequently. At most FQHCs, medical assistants (MAs) administer tobacco screenings and input results into the patients’ electronic health records (EHR). Based on those results, staff discuss treatment options, including medications, provider-mediated counseling, or referrals to an external or internal cessation program. If patients are unwilling to begin tobacco cessation, they are asked about it at another time. Many FQHCs refer patients to state programs like QuitWorks to connect with tobacco cessation counselors. As COVID-19 restrictions have lifted, some FQHCs have capitalized on the adoption of telehealth to offer hybrid health services - resuming in-person visits for services that require an office assessment, while continuing to offer telehealth visits when appropriate. One Care Coordinator saw this as a facilitator to tobacco cessation implementation because it “allows us to reach patients that we wouldn’t necessarily be able to reach.”

### Factors that Influence Implementation

#### Intervention Characteristics

A few staff members noted the overall ease of conducting tobacco use screenings and counseling patients as part of EHR-mediated tobacco cessation interventions. Typically, there are templates within the EHR system that will prompt the user with standardized documentation and referral tools. It may require maneuvering through multiple boxes or pop-ups. Participants said understanding and using the EHR was not difficult. However, some noted that too many steps deterred providers from following the intervention because it became burdensome to already overworked staff with too little time. As one staff member mentioned, “providers are just tired of clicking.” The QI Manager at another FQHC reflected on the complexity of training needed for implementing these interventions:

The screening part and the counseling part is easy for MAs and providers. What is hard is training people and retraining people… Until the end of last year, we only had one EMR trainer, she was the only one who was responsible for retraining the existing staff and training the new staff on how to document these screening tools. That is something that has been challenging for us, because one person could only do so much.

The core content of the interventions were not difficult by themselves; the steps surrounding the interventions, like training staff members and fatigue towards multiple pop-up reminders, made implementation complex.

#### Inner Setting

Various inner setting factors influence how tobacco cessation interventions are implemented. FQHCs rely on internal infrastructures like EHRs to facilitate intervention delivery. MAs enter data into pop-ups within medical records if patients need tobacco cessation interventions. These pop-ups remind providers to address the patient’s tobacco use. Staff also reflected on the need for enough time to implement tobacco cessation interventions. The short visit times (averaging 10–15 minutes) did not typically allow for comprehensive tobacco cessation screening and counseling. A Population Health Manger explained:

We’re faced with limited time during a visit. Often 15 minutes is not enough to cover a lot of things. One of the things that our providers sometimes are unclear about or there’s not enough guidance is how many minutes is required to count for counseling. Is it 5? Is it 10 minutes? If it’s 10 minutes, it’s almost the entire visit.

Some providers need to prioritize more acute medical concerns over preventative measures, unless “[smoking] was something that was interfering with a patient’s daily life”. This issue of short clinic visit time was addressed by utilizing non-physician staff at one FQHC, allowing providers to refer patients to the clinical pharmacists, who had much more appointment availability and time to counsel patients on tobacco cessation intervention options. Another participant mentioned a past dedicated tobacco cessation counselor who no longer works at the FQHC. Now, instead, their newly-established asthma clinic has a dedicated healthcare team comprised of a provider, nurse, and community health worker (CHW) that also address smoking. They said, “we’re starting to see staff and providers referring patients to this team. That’s the biggest thing, having a designated team that has a specialized focus on this area is going to be really helpful.”

Participants reported MAs and other non-physician staff members provide invaluable support for intervention activities. MAs typically conduct tobacco screenings and update the EHR to prompt reminder messages for providers, and in some cases, provide some tobacco cessation counseling. Without them, staff members have seen providers become overwhelmed during clinic visits and “sometimes might skip that screening or that questionnaire asking about tobacco status if [providers] feel [they] don’t have the time.”. Thus, expanding the responsibility of tobacco cessation EBI delivery to include more of the healthcare team can ensure the time and attention needed to positively affect the implementation of those EBIs.

#### Characteristics of Individuals

Beyond the need for engaging a multidisciplinary team, participants reflected on the characteristics of individuals that have influenced the implementation of tobacco cessation EBIs. A few participants spoke about the need for healthcare providers to be motivated to do the EBI and to, in turn, be encouraging in their interactions with patients. To effectively discuss cessation EBIs, providers must also know how to assess patient readiness. A Vice President shared:

If you have a provider who’s sensitive to the concept of assessing readiness and has either some motivational interviewing techniques or, from their provider interviewing skills, has an understanding of assessing that readiness, that’s going to be a better opportunity on the provider side.

Participants reported the loss of MAs, nurses, doctors, and other staff members. These shortages required others to fill roles or expand responsibilities. With fewer MAs, providers may have had to take on more of the tobacco screening or the remaining MAs may have had to increase their workload. In either case, staffing issues like this impacted the way tobacco cessation EBIs were implemented. As one QI Manager reflected, “sometimes, when we do not have MAs, providers have to [do] all the work… Therefore, sometimes providers might skip doing the tobacco screening, because there is more that they need to work on at that visit with the patient.”

#### Outer Setting

Common outer setting factors that affected the implementation of smoking cessation interventions included HRSA FQHC requirements and socioecological characteristics of the community. The FQHC status influences the implementation of tobacco cessation EBIs. FQHCs monitor tobacco use as a part of their required HRSA standard quality metrics. As one leader described, “tobacco use assessment intervention is one of the standard quality metrics for FQHCs. We’ve been thinking about it for the entire 15 years that I’ve been at the health center.”

Many of the FQHCs attributed their focus toward tobacco cessation interventions to requirements from different grants they had received. Some grants had explicit objectives to include smoking cessation interventions. A Director of Public Health Programs reported:

We have a grant from [the Department of Public Health] … that’s focused on asthma prevention and control, but one of the objectives is on smoking cessation, so there are certain activities that we do to meet those grant requirements… related to being able to connect people and provide linkages to community-based support for smoking cessation.

Some staff members spoke of the need for tobacco cessation interventions because of the prevalence of tobacco use in the neighborhoods in which their FQHCs are situated. A QI Manager reflected on the community influences on the need for screening: “Tobacco screening is something I am strongly advocating. Even though the standard is to begin at 13, or in some cases 15, I always say start at 12. There is a lot of smoking, tobacco use, and vaping that’s happening in the community, especially for high school kids.”

### Nutrition And Physical Activity

#### Implementation Process

As seen in the survey results, most staff described offering EBIs that target nutrition and physical activity. These included the Diabetes Prevention Program (DPP), Chronic Disease Self-Management Program (CDSMP), and Diabetes Self-Management Program (DSMP). Staff reported these classes are typically led by a range of staff members, including CHWs. One FQHC operates a Wellness Center dedicated to nutrition and physical activity, which offers programs in various languages. Almost all participants spoke about strategies that have limited evidence for effectiveness on chronic disease prevention. For example, some staff described delivering nutrition or physical activity classes that were developed in house with few details how evidence-based content was incorporated and no mention of evaluation. Many FQHCs had partnerships with local food banks, food pantries, and farmers’ markets. They encouraged patients to use these resources to eat healthier foods, with the primary objective of addressing food insecurity.

### Factors that Influence Implementation

#### Intervention Characteristics

To meet the needs of a multilingual patient population, some FQHCs adapted nutrition and physical activity EBIs to be delivered in languages other than English. This was challenging as some FQHCs did not have the needed language capacity. The Director of Public Health Programs at one FQHC also identified that to administer evidence-based CDSMP classes, staff had to obtain specific training and certification. They described that this made holding these classes in different languages even more challenging:

For the CDSMP and DSMP classes, those right now are only offered in English and Spanish because there are specific requirements for being able to facilitate those classes in other languages. There’s a whole lengthy set of requirements that we have to comply with in order to have staff certified to lead those classes.

Most of the nutrition and physical activity EBIs were conducted in person at FQHCs. Some FQHCs were able to successfully adapt these programs to virtual or hybrid offerings amidst the pandemic and provided tablets as needed so that patients could continue to access these programs. According to most participants, the interventions themselves were not difficult to implement. However, it was challenging to recruit patients for these EBIs and keep them coming to the classes because of the time commitment. A Population Health Manager reported:

We had a couple of rounds on one of the chronic disease self-management programs through the Stanford Model a few years back. That was one of the offerings we had for our patients… It’s six weeks, two-hour classes. It’s very hard to recruit and retain patients in that program.

Like the challenges faced with implementing tobacco cessation EBIs, participants mentioned that the design of existing EMR documentation and referral functionality could be burdensome and complex for providers with already overwhelming workloads and short clinic visit times per patient. A Director of Performance Improvement worried that providers may be tempted to ignore such pop-ups, “these EMR prompts sometimes run the risk of overload or getting ignored because there are too many of them”.

#### Inner Setting

The workflow of nutrition and physical activity interventions at most FQHCs starts from the moment the patient interacts with front-facing staff like MAs. These staff members take weight measurements, blood pressure, and other health indicators and input them into the patient’s EHR. Similar to tobacco cessation interventions, EHRs are programmed to alert or remind providers to address nutrition and physical activity if a health metric falls within a certain range (e.g., a BMI > 30 may trigger a pop-up).

Nutritionists, MAs, and other dedicated staff members are further influential in implementation as key actors that promote and actively prioritize nutrition and physical activity EBIs. A clinical nutritionist at one FQHC contributes to education for patients who may still have follow-up questions not addressed in their short primary care visit. A Population Health Manager described a unique collaboration: “working alongside with the nutritionist, our clinical pharmacist is very well utilized. She’s an add-on for our nutrition program that can also provide some more education.”

#### Characteristics of Individuals

Overall, staff reported positive attitudes towards the nutrition and physical activity EBIs. A Clinical Assistant described the DPP: “it’s an amazing program. Everybody should do it [laughs]. It’s well-being for the patient…The situation that we’re living now, it makes it a little bit harder, but it’s not impossible. Where there’s a will, there’s a way.” The Care Coordinator at another FQHC described the importance of having dedicated personnel with specialized skills support delivery: “I do think our nutritionist is a good resource, and that she’s very useful, and that patients enjoy seeing her.” However, the Quality Manager at another FQHC reflected that having a skilled team to help with EBIs does not matter if providers are not aware of their existence. They said:

We actually have a pretty decently-sized case management team. I think there’s five of them. If the providers aren’t utilizing them or don’t know how, then it doesn’t really matter. We are trying to build up a strong program where everyone understands how to utilize that team and how to refer to them. Same with the nutritionists.

Health care providers must know of the resources within their own workplace so that they can refer patients for nutrition and physical activity care as needed.

#### Outer Setting

Staff recognize that they serve a diverse range of patients with varying economic and educational circumstances, as well as different language needs. Patient needs are understood through interactions with front-facing staff, word of mouth, and a general survey of the population. They address these needs by tailoring nutrition or physical activity classes to be culturally appropriate and held in languages spoken by their patient population.

Influences in the outer setting, like grant and insurance requirements, help to prioritize some EBIs. A few FQHCs received the MDPH 1817 grant from the CDC (23) that supports programs and activities to prevent and manage type 2 diabetes. One FQHC received funding from the Healthy Living Center of Excellence, which they used to support reimbursement for patients who completed physical activity classes.

However, staff reported that grants can be transient, which makes resources for interventions fleeting at times. Some staff members remembered when they had more support, infrastructure, and resources for a particular intervention. When the grant ended, the intervention activities also ceased.

Implementing tobacco cessation and nutrition and physical activity EBIs at FQHCs depended on many factors, including the complexity of the intervention itself, the workflow and resources of the FQHC, the various key players supporting intervention delivery and referral, and policy and community influences outside the walls of the FQHC.

#### Partnerships

Few participants reported partnerships with community organizations for cancer prevention when prompted during interviews. Some said they made connections through membership in an Accountable Care Organization (ACO). For example, a staff member mentioned connecting to a community development corporation and an asthma support program through their ACO. Staff at other FQHCs attributed partnerships to the grants they have received. However, grant-facilitated partnerships have the potential to dissolve when the funding ends. A Patient Educator described how the end of funding impacted the implementation of EHR-mediated interventions at their FQHC:

We also receive[d] funding from the Prevention and Wellness Trust Fund… We did build a partnership with the agency who do[es] smoking cessation. We did have the workflow built by DPH… to refer patient[s] directly to our EMR system. When the funding end[ed] in 2018, we did not want to continue to do that workflow or that referral process because they don’t have anybody to manage that. We stop[ped], and we go back to the routine to do it manual[ly].

Some staff commented on collaborations or agreements with local gyms and farmers’ markets to promote nutrition and physical activity and recognized the value of partnering with community-based organizations to increase community awareness of FQHC resources available. However, partnership for delivery of EBIs was rarely discussed. Some staff showed interest in developing partnerships with community organizations in the future. Leveraging new partnerships fostered during the pandemic was mentioned by a QI Manager: “especially with COVID. We were lucky enough to form more relationships with our various community partners. That really is going to drive this”. However, the specifics of how FQHCs might partner for EBI delivery were unclear. A Director of Performance Improvement shared:

I always think we can benefit from working with community partners. I don’t think I know exactly what’s out there, what other health centers are doing… I would be interested in hearing about whether some of the other health centers have figured anything out. I’m sure that there’s ways that we could benefit from partnerships.

While most participants did not report active community partnerships for delivering of cancer prevention EBIs, Caring Health Center in Springfield stood out as a positive case example. Caring staff discussed partnerships at various levels of engagement, ranging from networking to exchange resources and share information to collaborating with people from local community organizations for EBI delivery. For example, staff from Men of Color Health Awareness and Ascentria Care Alliance co-facilitate nutrition and physical activity classes for patients at the FQHC. The FQHC is also well-connected with their local Public Health Institute from whom they have received tobacco cessation resources. A Caring Health Center leader described how they leverage the community relationships to connect patients with resources: “we have a whole directory of community-based resources and national helplines…that are focused on smoking cessation that our CHWs will connect people with as needed.”

Table 2 provides a comprehensive integration of qualitative and quantitative findings.

## Discussion

This mixed methods study of primary cancer prevention EBIs provides an exploration of nutrition, physical activity, and tobacco use interventions in Massachusetts FQHCs. All FQHCs surveyed offered evidence-based clinician-delivered screening and counseling, Quitline interventions, tobacco cessation medication, and diet/physical activity programs that were developed to prevent type 2 diabetes. Most FQHCs offered coach and mobile-delivered tobacco cessation counseling and social support physical activity interventions. Group tobacco cessation counseling was offered at only one-third of FQHCs. Although EBIs were available, they were rarely offered to most eligible patients. Follow up qualitative data explain the ways in which context impacts EBI implementation. We found multilevel factors influenced implementation across all intervention types – from the complexity of intervention trainings, to available visit time and staffing resources available in the inner setting, to motivation of clinicians, to external policies and incentives in the outer setting. While partnerships were described as valuable to many staff, only one FQHC (Caring) reported using partnerships for the referral and delivery of primary cancer prevention EBIs.

FQHCs excel at providing in-house preventive clinical care. This is, in part, because they are required by HRSA to collect Uniform Data System quality of care measures for practices like colorectal and cervical cancer screening annually to assess impact and drive QI (2). However, adoption and penetration data revealed EBIs that depend on collaboration outside of the office visit had less uptake and patient reach. This is likely due to the need for more focused expertise, patient time, and coordination required for behavior change intervention like group tobacco cessation counseling and social support physical activity programs. FQHCs may not always have the infrastructure (e.g., staffing, training capacity, ongoing funding) to deliver cancer prevention EBIs in a sustainable or standardized way. Partnerships with community organizations could increase delivery of these more comprehensive EBIs when staff capacity to meet patient volume and FQHCs workflows and visit times are not compatible with delivery. While only one FQHC described working with community partners to deliver EBIs, this was not for a lack of interest among others. There are a wealth of partnership opportunities in the communities where FQHCs are located, but the challenge comes with having the resources and team dedicated to operationalizing these partnerships through collaborative agreements and develop organized bi-directional referral systems (13).

Strengths of this study include its mixed methods approach for developing a comprehensive understanding of EBI implementation – with only one type of data, we would have an incomplete picture of the state of primary cancer prevention in FQHCs. For instance, qualitative findings revealed the contextual factors that help explain the limited reach of EBIs even when adoption was high. Situating the study within the ISCCCE infrastructure, including qualitative interviews, and engaging FQHC co-authors ensured that the data collected and reported centered the perspectives of FQHC staff. However, the study is not without limitations. We collected data at the height of the COVID-19 pandemic, which led to lower response rates than expected. Although questions asked staff to report on practices prior to the pandemic, answers may have been impacted by changes in FQHC offerings. Reports of adoption and penetration are based on self-report survey data that could be biased or incomplete and are limited to sites in Massachusetts; future research should compare these estimates to data collected through EHR systems across a broader geographic area for more precise and generalizable results. Situating the FQHC staff perspectives on the CDC continuum (11, 12), clinical-community linkages were typically viewed as “partnerships” only when they entailed collaboration. In fact, several interviewees reported they did not consider less intensive information exchange and coordination like referral as partnerships. Thus, the future research on clinical-community partnership warrants should use more precise language.

Our findings highlight key considerations for research and practice. Future research should explore the nuances between FQHC-based EBI delivery vs. services offered via referral building on studies of social determinants of health referral adoption (24). An extension of this implementation science research would be to determine the distinction between offering and actual utilization of cancer prevention EBIs. Our findings and the minimal body of research we build upon (6–9), highlight the importance of increasing evaluation of EBIs and homegrown programs within FQHC and community settings in order to better understand what strategies are most feasible, accessible, and effective for addressing cancer prevention.

## Conclusion

There is great interest in implementing primary prevention EBIs in FQHCs, but stable staffing and funding is required to successfully reach all patients. Relying on grant funding means these essential services can be treated as optional and program churn can impact providers’ knowledge of what is available at any given time. FQHCs need support to offer complex EBIs that have more limited adoption and often necessitate adaptation for language and local context. FQHC staff are enthusiastic about the potential of community partnerships to foster improved implementation – defining the range of possibilities for these linkages and providing training and support to build these relationships to support evidence-based prevention will be key to fulfilling that promise.

## Figures and Tables

**Table 1:** Proportion of patients offered or referred to EBIs in Massachusetts-based FQHCs estimated by staff (N = 34)

Evidence-based intervention	None	Not too many	Some	Many	Most or all
***Tobacco Use***					
Clinician-delivered screening practices (e.g. Ask, Advice, Refer (AAR), 5 A’s) (N = 33)	0.00%	12.12%	27.27%	27.27%	33.33%
Prescription of tobacco cessation medications (N = 34)	0.00%	2.94%	38.24%	41.18%	17.65%
Clinician-delivered individual tobacco cessation counseling (N = 33)	0.00%	6.06%	36.36%	48.48%	9.09%
Coach or counselor delivered individual tobacco cessation counseling (N = 32)	21.88%	28.13%	18.75%	18.75%	12.50%
Group tobacco cessation counseling (N = 32)	68.75%	15.63%	3.13%	6.25%	6.25%
Quitline interventions (e.g., Quitworks, 1–800-Quit-Now) (N = 33)	3.03%	18.18%	36.36%	24.24%	18.18%
Mobile phone-based cessation interventions (e. g. SmokefreeTXT or other text message interventions) (N = 32)	50.00%	25.00%	9.38%	12.50%	3.13%
Nutrition & Physical Activity					
Diet and physical activity promotion program to prevent type 2 diabetes among people who are at risk (e.g. Diabetes Prevention Program) (N = 33)	3.03%	12.12%	27.27%	33.33%	24.24%
Social support physical activity program focused on building, strengthening, and maintaining social networks for behavior change (e.g. buddy system, walking groups) (N = 32)	12.50%	31.25%	28.13%	15.63%	12.50%
Worksite nutrition and physical activity program for community health center staff (N = 34)	32.25%	20.59%	20.59%	5.88%	20.59%

**Table 2:** Integration of quantitative and qualitative findings on EBIs for primary cancer prevention among Massachusetts FQHCs

	Tobacco Use	Nutrition & Physical Activity
FQHC adoption	100% FQHCs offered clinician-delivered screening (e.g., 5As) and counseling, Quitline interventions, and tobacco cessation medication 81% FQHCs offered coach-delivered tobacco cessation counseling 63% FQHCs offered mobile phone-based cessation interventions 36% FQHCs offered group tobacco cessation counseling	100% FQHCs offered diet and physical activity programs to prevent type 2 diabetes 94% FQHCs offered social support physical activity programs 81% FQHCs offered worksite nutrition and physical activity programs for CHC staff
Patient penetration	1/3 staff reported most/all eligible patients offered clinician-delivered screening (e.g., 5As) <1/4 staff reported most/all eligible patients offered all other tobacco use interventions 1/2 staff reported none/few eligible patients offered coach-delivered tobacco cessation counseling 3/4 staff reported none/few eligible patients offered mobile phone-based cessation interventions	1/4 staff reported most/all eligible patients offered diet and physical activity programs to prevent type 2 diabetes <1/4 staff reported most/all eligible patients offered all other nutrition and physical activity interventions >1/3 staff reported none/few eligible patients offered social support physical activity programs
Key factors influencing implementation	Intervention characteristics: Complexity of trainings and referrals Inner setting: IT infrastructure (e.g., EHR) Work infrastructure (e.g., visit time, workflows) Relative priority Available resources (e.g., funding, staff dedicated to implementation) Characteristics of individuals: Beliefs about EBI (e.g., motivation to deliver) among clinical staff Knowledge and skills to assess patient readiness Staffing issues (e.g., shortages) Outer setting: External policies and incentives (e.g., FQHC HRSA requirements, funding requirements) Socioecological characteristics of community	Intervention characteristics Complexity of recruitment, retention, and training Adaptability based on culture and language Inner setting: Work infrastructure (e.g., visit time) Available resources (e.g., staff dedicated to implementation) Characteristics of individuals: Knowledge and beliefs about EBI (e.g., motivation to deliver, awareness of resources available) among clinical staff Outer setting: Patient needs (e.g., language access) External policies and incentives (e.g., insurance and funding requirements)
Partnerships	Only one CHC reported partnerships for tobacco EBIs – partnerships primarily for referrals to outside resources	Only one CHC reported partnerships for nutrition and physical activity EBIs – partnerships for co-delivery of evidence-based programs at CHC

## Data Availability

The data collected and analyzed during the current study are available from the corresponding author on reasonable request and will be shared in accordance with Cancer Moonshot funding policies.

## Abbreviations

CDC: Centers for Disease Control and Prevention
CDSMP: Chronic Disease Self-Management Program
CFIR: Consolidated framework implementation research
CHW: Community Health Worker
DPP: Diabetes Prevention Program
DSMP: Diabetes Self-Management Program
EBI: Evidence-based intervention
EHR: Electronic health record
FQHC: Federally Qualified Health Centers
HRSA: Health Resources and Services Administration
ISCCCE: Implementation Science Center for Cancer Control Equity
MA: Medical assistant
